# MiRNAs与EGFR-TKIs继发性耐药机制的研究进展

**DOI:** 10.3779/j.issn.1009-3419.2015.12.08

**Published:** 2015-12-20

**Authors:** 明 王, 震宇 孙, 礼年 黄

**Affiliations:** 1 233000 蚌埠，蚌埠医学院 Bengbu Medical College, Bengbu 233000, China; 2 233000 蚌埠，蚌埠医学院第一附属医院呼吸与危重症学科 Department of Respiratory and Critical Care Medicine, the First Affiliated Hospital of Bengbu Medical College, Bengbu 233000, China

**Keywords:** 肺肿瘤, MiRNAs, EGFR-TKIs, 继发性耐药, Lung neoplasms, MiRNAs, EGFR-TKIs, Secondary resistance

## Abstract

肺癌是癌症致死率最高的疾病，关于这个疾病的发生机制已得到部分阐明，其中表皮生长因子受体（epidermal growth factor receptor, EGFR）信号通路研究最为深入，在肺癌的发生中起着至关重要的作用。而有效地抑制EGFR信号通路的药物已用于非小细胞肺癌（non-small cell lung cancer, NSCLC）的靶向治疗中，伴有EGFR基因突变的患者使用EGFR酪氨酸激酶抑制剂（EGFR-tyrosine kinase inhibitors, EGFR-TKIs）治疗后获得不错的临床收益，但大部分患者在使用该药治疗10个月后出现耐药现象。MiRNAs（microRNAs）是一种非编码蛋白的RNA，参与转录后水平基因的表达调控。越来越多的研究发现miRNAs与EGFR-TKIs继发性耐药有关，miRNAs可作为逆转EGFR-TKIs耐药及评估EGFR-TKIs有效性的生物指标。本文就NSCLC中miRNAs与EGFR-TKIs继发性耐药机制之间的相关性研究进展做简要的综述。

## Abstract (en)

Lung cancer is a leading cause of cancer mortality worldwide. Several molecular pathways underlying mechanisms of this disease have been partly elucidated, among which the epidermal growth factor receptor (EGFR) pathway is one of the well-known signaling cascades that plays a critical role in tumorigenesis. The strategies to effectively inhibit EGFR signaling pathway have been used in non-small cell lung cancer (NSCLC) targeted therapy. Patients with *EGFR* mutations benefit from EGFR-tyrosine kinase inhibitors (EGFR-TKIs) treatment. However, most of TKIs-treated patients eventually suffer drug resistant after 10-month treatments. MiRNAs (microRNAs) is a non coding RNA and protein involved in regulating gene expression in the transcription level. More and more studies have found that miRNAs are correlated with EGFR-TKIs secondary resistance. MiRNAs may serve as novel targets to circumvent the resistance and promising predictive biomarkers for EGFR-TKIs. In this paper, we reviewed briefly advanced research on miRNAs and EGFR-TKIs secondary resistance in NSCLC.

肺癌是世界上最常见的恶性肿瘤之一，其死亡率位于恶性肿瘤的首位。目前约仅有30%的患者获得早期诊断，并获得手术治疗，对于失去手术机会的晚期患者5年生存率小于15%^[1]^。肺癌包括分为两种类型：非小细胞肺癌（non-small cell lung cancer, NSCLC）和小细胞肺癌（small cell lung cancer, SCLC），两者各占比例分别约为87%和13%。目前含铂双药化疗方案仍是晚期NSCLC推荐的一线方案，但随着对NSCLC发生、发展分子机制的了解，目前已有一些靶向药物[如表皮生长因子受体酪氨酸激酶抑制剂（epidermal growth factor receptor tyrosine kinase inhibitors, EGFR-TKIs）]应用于临床，并获得不错的临床收益。

EGFR是由N-端胞外的配体结合区域、疏水的跨膜区域、细胞内的催化区域（含酪氨酸激酶活性）组成^[2]^。EGF结合EGFR形成异二聚体，激活并磷酸化胞内酪氨酸激酶，继而激活一系列下游信号通路，如JAK-STAT、Ras-Raf-MAPK、PI3K-Akt-mTOR，参与细胞增殖、分化的调节。而EGFR信号通路的异常调节可引起肿瘤的发生、增殖、侵袭和转移，目前靶向该通路的有单克隆抗体（cetuximab、panitumumab）和激酶抑制剂（gefitinib、erlotinib、afatinib），这些药物毒副反应较轻，明显改善了NSCLC患者的生存质量和生存期。但NSCLC患者中只有部分伴有*EGFR*突变阳性的患者受益，且这些药物最终还可产生继发性耐药，出现病情进展。目前，*EGFR*突变作为评估TKIs药物有效性的生物学标志，但实际临床样本*EGFR*突变检测存在一定的技术难题，PCR技术的检测假阳性率较高，而作为金标准的EGFR测序阳性率较低。

目前研究发现的EGFR-TKIs继发性耐药机制有：①细胞内*EGFR*基因受体结合区突变（T790M）：增强了催化区域与ATP的结合能力，抑制了其与TKIs的结合; ②Met基因扩增：通过激活不依赖EGFR的ERBB3的磷酸化作用以及下游区的PI3K-Akt-mTOR通路; ③胰岛素样生长因子1受体（insulin-like growth factor 1 receptor, IGF-1R）的激活：通过激活PI3K/AKT信号通路引起TKIs的耐药; ④EGFR扩增7号染色体的缺失; ⑤第10号染色体磷酸酶和张力蛋白同源缺失基因（*PTEN*）基因突变; ⑥表型转化：NSCLC转化为SCLC、上皮间质转化（epithelial-to-mesenchymal transition, EMT）等。针对耐药问题，microRNAs（miRNAs）与EGFR信号通路相关性的研究已成为热点之一。MiRNAs是一类由内源基因编码的长度约22个核苷酸的非编码小分子RNA，参与转录后水平基因的表达调控。MiRNAs几乎参与了所有细胞生物学行为的调控，并在肺癌的发生、发展中发挥重要作用^[3]^，可起到致癌基因或抑癌基因的作用。MiRNAs有望成为改善EFGR-TKIs耐药的新靶点，为治疗NSCLC开拓新的方向。

## 1 MiRNAs与EGFR信号通路之间的调节关系

越来越多的试验证实miRNAs与EGFR的表达、*EGFR*突变状态及信号通路的激活息息相关。Dacic等^[4]^报道miR-155只在EGFR/KRAS阴性的肺腺癌中高表达，miR-25只在EGFR阳性的腺癌中高表达，miR-495只在KRAS阳性的腺癌中高表达，而let-7g在以上三种类型腺癌中均呈低表达，特别是EGFR/KRAS阴性。

Liu等^[5]^研究发现miR-133b在NSCLC组织中低表达（*P*=0.002），miR-133b和EGFR mRNA的表达量呈负相关（*r*=-0.71, *P* < 0.001）; 将miR-133b转染入PC-9和A549细胞，发现miR-133b可抑制EGFR、AKT、ERK1/2的磷酸化，从而抑制EGFR信号通路，诱导细胞凋亡、抑制细胞侵袭，并增加对EGFR-TKIs的敏感性。Wang等^[6]^研究表明，miR133a在NSCLC组织及细胞中呈低表达，miR133a通过抑制胰岛素样生长因子受体（insulin-like growth factor receptor, IGF-R）、转化生长因子β-R1（transforming growth factor-β receptor type-1, TGFβ-R1）和EGFR来调节AKT信号通路，最终抑制肿瘤细胞的增殖、侵袭。MiR133a与总生存期有关，miR133a高表达是预后良好的指标。因此，miR133a不仅可作为判断预后的指标，也是NSCLC未来潜在治疗靶点。

Yoo等^[7]^研究多种肺癌细胞株（WI-38、A-13、A549、HCC-1588、NCI-H596）及15例肺癌组织，发现miR-9500呈低表达，miR-9500通过抑制AKT1（EGFR信号传导过程中关键受体）来抑制肿瘤的增殖、侵袭和转移。另外，同样作用于AKT，起到抑癌作用的还有miR-153^[8]^，然而miR-200^[9]^起着致癌基因功能，AKT抑制剂可用于治疗miR-200依赖性的肺癌患者。

以上试验揭示了miRNAs对EGFR信号通路的调节作用，相反，EGFR异常表达或突变同样可影响miRNAs的表达。MiRNAs和EGFR信号通路之间形成反馈调节，参与肺癌的发生、发展。Guo等^[10]^研究发现，在EGFR突变型或野生型的肺癌细胞株（H1975、H1650、A549及H292）中miR-145表达下调，且miR-145和p-EGFR呈负相关关系（*P* < 0.05），EGFR信号通路的激活可引起miR-145的表达下调; 应用EGFR抑制剂AG1478作用于H1975细胞（EGFR突变型）、A549细胞（EGFR野生型）、正常细胞株BEAS-2B，可逆转EGFR信号通路对miR-145的负性调节。

## 2 MiRNAs与EGFR-TKIs继发性耐药的关系

对EGFR信号通路的深入研究促进了EGFR-TKIs的发展，目前应用于临床的有gefitinib、erlotinib和afatinib。但因为继发性耐药，使EGFR-TKIs临床受益受限，其中70%是由于*EGFR*基因二次突变（T790M、c-MET扩增）。

### 2.1 MiRNAs与EMT

EMT发生过程中，上皮特异性标志物（E-钙粘蛋白等）表达下调，间质特异性标志物（波形蛋白等）表达上升，使上皮细胞失去细胞极性及与基膜连接的功能，获得细胞运动性、细胞迁移和侵袭等相关的间质功能，使上皮细胞来源的恶性肿瘤细胞获得迁移和侵袭能力，甚至参与EGFR-TKIs耐药等重要生物学过程。

近年来研究发现，miRNAs在EMT过程中充当关键调节者，且不同的miRNAs通过不同的靶点调控EMT的发生。Xia等^[11]^研究发现，在NSCLC组织中，miR-638表达明显下调，miR-638的表达下调可致E-钙粘蛋白的减少，纤粘蛋白、波形蛋白和Snail的上升，而将携带有miR-638的质粒转入NSCLC细胞系，发现E-钙粘蛋白上升，纤粘蛋白、波形蛋白、Snail降低。因此，miR-638的表达下调可通过诱导EMT参与NSCLC的增殖和侵袭。Seol等^[12]^研究发现，在NSCLC组织及细胞中，miR-373表达下调，将携带有pre-miR-373的质粒转染入A549和Calu-6细胞，波形蛋白、N-钙粘蛋白、纤粘蛋白表达下调，IRAK2和LAMP1表达下调; 将靶向IRAK2和LAMP1的siRNAs转染入上述两种细胞，间质标志物同样出现表达下调，两种细胞的侵袭、转移明显受到抑制。因此，miR-373可通过靶向IRAK2和LAMP1抑制EMT，miR-373/IRAK2/LAMP1轴可成为NSCLC新的治疗靶点。Suh等^[13]^研究发现，在NSCLC中，miR-30c表达下调，波形蛋白、纤粘蛋白水平升高和E-钙粘蛋白水平下调，miR-30c可以通过靶向波形蛋白、纤粘蛋白调控TGF-β诱导的EMT，此外，还可通过靶向MTDH和HMGA2抑制NSCLC的转移。因此，miR-30c的表达水平可能作为NSCLC转移的标志物，增加miR-30c的表达可能有助于延缓病情进展、抑制肿瘤转移。miR-30c与EMT的发生关系同样也得到其他学者的证实^[14]^。Li等^[15]^研究发现，在NSCLC中mi-R-148a呈低表达，ROCK1呈高表达; 将携带有miR-148a的质粒转染入H1299细胞，发现ROCK1表达下调，E-钙粘蛋白水平升高，波形蛋白水平下降，细胞的侵袭性减弱; 而si-ROCK1的使用同样会引起上述上皮、间质标志物的改变，即提示了miR-148a可通过调节ROCK1的表达抑制EMT的发生，抑制NSCLC的侵袭、转移。同样，在NSCLC中低表达，表现为抑癌基因功能，抑制肿瘤增殖、侵袭、转移的还有miR-145、miR-135a、miR-132、miR-33a、miR-125b、miR-146a。miR-145通过靶向Oct4参与调节EMT的发生^[16]^;miR-135a通过靶向KLF8调节EMT^[17]^;miR-132通过靶向ZEB2抑制EMT的发生^[18]^;miR-33a通过靶向Twist1调节EMT的发生^[19]^;miR-125b在紫杉醇耐药的NSCLC细胞株A549、H460中呈低表达，可通过靶向Sema4C调节EMT的发生^[20]^;miR-146a不仅可以通过靶向胰岛素受体底物-2（insulin receptor substrate 2, IRS-2）抑制EMT的发生，还能使EGFR-TKIs耐药的NSCLC患者恢复对gefitinib的敏感性^[21]^。

而某些miRNAs在NSCLC中表现为致癌基因，能诱导EMT的发生，促进肿瘤的转移和侵袭，导致其对EGFR-TKIs的耐药。Cao等^[22]^研究发现，在NSCLC细胞株（A549、LC-2/ad、ABC1、PC1、SQ5）中，miR-23a和Smad2/3表达上调。将TGF-β1刺激A549细胞，导致miR-23a表达明显升高，E-钙粘蛋白表达下调，N-钙粘蛋白表达上调。将siRNAs干扰Smad2/3的表达，可引起miR-23a表达下调，TGF-β1/Smad信号通路可调节miR-23a的表达。将miR-23a抑制剂转染入A549细胞中，随后再用TGF-β1刺激该细胞，发现E-钙粘蛋白仍可表达，而N-钙粘蛋白表达微弱，miR-23a抑制剂可部分抑制TGF-β诱导的EMT。将Pre-miR-23a转染入A549细胞中，引起E-钙粘蛋白的下调、波形蛋白的上调。因此，miR-23a可通过靶向E-钙黏蛋白（cadherin）基因（*CDH1*基因）调节TGF-β1诱导的EMT，导致对gefitinib的耐药。Xu等^[23]^研究发现，在过表达hsa-miR-191的人气道上皮细胞中，E-钙粘蛋白表达下调，N-钙粘蛋白、波形蛋白表达上调。将miR-191抑制剂转染入人气道上皮细胞中，发现N-钙粘蛋白、波形蛋白的表达下调，E-钙粘蛋白的表达上调，抑制EMT的发生。

增加抑癌基因型miRNAs和减少致癌基因型miRNAs的表达或许可成为NSCLC治疗的新靶点，起到抑制肿瘤侵袭和转移、增加对EGFR-TKIs敏感性的作用。

### 2.2 MiRNAs与*PTEN*基因缺失

研究发现，*PTEN*基因缺失可导致EGFR-TKIs继发性耐药的产生。Shen等^[24]^发现，NSCLC组织中miR-21呈高表达（78.7%, 37/47），PTEN蛋白呈低表达（72.3%, 34/47），miR-21的表达与PTEN呈负相关; 46例NSCLC患者在用TKI（gefitinib或erlotinib）治疗后，相对疾病稳定（stable disease, SD）组和部分缓解（partial response, PR）组，疾病进展（progressive disease, PD）组miR-21升高，PTEN降低; 相对PC9细胞（gefitinib敏感），PC9/GR细胞（gefitinib耐药）的miR-21表达约为3.70倍，*PTEN*基因缺失，AKT和ERK均呈激活状态; 先将miR-21转染入PC9细胞，发现其对gefitinib的敏感性降低，再将PTEN转染入PC9细胞，发现PTEN可恢复细胞对gefitinib的敏感性; 将miR-21抑制剂转染入PC9/GR细胞，发现可恢复细胞对gefitinib的敏感性。因此，抑制miR-21的表达可能会逆转EGFR-TKIs耐药。关于miR-21与PTEN之间的调控关系分别在肺鳞癌、腺癌中也得到证实^[25, 26]^。Wang等^[27]^研究发现，miR-29b在NSCLC组织中表达下调，将携带有miR-29b慢病毒转染入A549细胞中，MMP2、PTEN mRNA表达下调，细胞的增殖、侵袭、转移能力减弱。携带有miR-29b抑制剂的慢病毒转染入H460细胞，MMP2、PTEN mRNA表达上调，细胞的增殖、侵袭、转移能力增强。因此，miR-29b通过直接靶向MMP2和PTEN抑制NSCLC的侵袭和转移，miR-29b可作为延缓NSCLC转移、改善EGFR-TKIs耐药的治疗靶点。MiRNA-10a在NSCLC组织中明显高表达，在高转移及低转移特性的NSCLC细胞（H1299、SPC-A-1sci、SPC-A-1、H358）中，miRNA-10a与PTEN的表达呈反比关系，miR-10a通过PTEN/AKT/ERK信号通路促进肿瘤的侵袭^[28]^。

### 2.3 MiRNAs与*c-Met*基因扩增

在NSCLC治疗过程中，miRNAs可通过调节*c-Met*基因，参与EGFR-TKIs继发性耐药。Zhou等^[29]^研究发现，miR-34a可能通过调控*Met*基因，克服EGFR-TKIs耐药。在HCC827、PC-9细胞（*EGFR*基因19号外显子缺少）中miR-34a高表达，Met低表达; 用HGF诱导产生gefitinib耐药细胞株（HCC827/GR、PC-9/GR），发现其中miR-34a低表达，Met高表达; 将miR-34a转染入HCC827/GR、PC-9/GR细胞，发现Met mRNA和蛋白水平下调; miR-34a单一治疗能减少Met的表达，但不能抑制EGFR的磷酸化及下游信号通路（PI3K/Akt信号通路）的激活，因而诱导HCC827/GR、PC-9/GR细胞凋亡的效果不佳; 而miR-34a联合gefitinib治疗可抑制90%以上的Met磷酸化，导致下游的Akt和ERK1/2磷酸化受到影响，可诱导HCC827/GR细胞的大量凋亡。Zhou等^[30]^发现miR-130a在Pc9细胞（gefitinib敏感）中高表达，而Met低表达; 将miR-130a转染入Pc9 GR细胞（gefitinib耐药），发现Met蛋白表达下调; miR-130a可通过靶向Met逆转gefitinib耐药，增强gefitinib诱导的肿瘤细胞凋亡。

靶向MET、起抑癌基因功能的还有miR-409-3p、miR-206、miR-200a。MiR-409-3p^[31]^在肺腺癌组织中低表达，可通过靶向Met抑制Akt信号通路，从而抑制肿瘤的增殖、侵袭。MiR-409-3p与pTNM分期、淋巴结转移有关，是判断预后的独立的指标。在NSCLC组织中，MiR-206^[32]^低表达，MET的3´UTR的484-508、796-823以及BCL2的3´UTR的24034-4057是miR-206的直接靶点，从而诱导肿瘤细胞的凋亡。MiR-200a^[33]^在NSCLC中低表达，可通过靶向EGFR和c-Met抑制肿瘤的侵袭和转移，提高耐药细胞株对gefitinib的敏感性。这意味着，miR-200a作为治疗靶点，可适用于EGFR-TKIs耐药的NSCLC患者。

## 3 MiRNAs评估EGFR-TKIs的有效性

越来越多的研究发现miRNAs可作为评估EGFR-TKIs有效性的标志物。Li等^[34]^研究发现在原发性TKIs耐药的NSCLC细胞株（A549、H1299、H23、H460、H1975）中，miR-200c呈低表达，150例NSCLC患者（73例*EGFR*突变型、66例EGFR野生型、11例未知）接受gefitinib治疗，客观缓解率（objective response rate, ORR）为34.7%，疾病控制率（disease control rate, DCR）为64.7%;其中伴有miR-200c高表达的EGFR野生型患者的临床受益明显优于低表达者，DCR（57.7% *vs* 27.5%, *P*=0.014），无疾病进展生存期（progression-free survival, PFS）（5.0个月*vs* 1.2个月，*P*=0.001），总生存期（overall survival, OS）（9.6个月*vs* 5.0个月，*P*=0.037），但在*EGFR*突变型患者miR-200c的表达对患者的PFS、OS、ORR无明显影响。因此，miR-200c可能用于判断EGFR野生型的NSCLC患者对EGFR-TKIs敏感性的指标。Shen等^[35]^研究了201例伴有EGFR突变型的NSCLC患者服用gefitinib的情况，发现其中miR-21低表达的患者OS明显改善（28.3个月*vs* 24.4个月，*P*=0.004, 5），但miR-21高表达的患者表现出对gefitinib更好的反应率（73.7% *vs* 30.7%, *P* < 0.001），miR-21可作为评估gefitinib敏感性的独立标志物。EGFR负性调节因子Mig6是miR-200的靶点，体内试验证实该Mig6/miR200的比值与erlotinib敏感性成反比关系。因此，Mig6/miR200可作为评估EGFR-TKIs敏感性的可信赖指标^[36]^。

## 4 讨论与展望

与传统的化疗相比，肺癌的靶向药物治疗具有方便、毒副作用轻的优点，但后期会出现耐药问题。MiRNAs是基因表达的调控者，通过精密、复杂的信号传导网络调控人类的生命活动。因此miRNAs异常可引起肿瘤耐药相关通路中基因表达水平的改变，从而导致耐药。目前针对miRNAs与EGFR信号通路之间调控关系的研究已取得部分成果，促进了miRNAs在肺癌的治疗进展，如let-7和miR-34联合治疗可协同增加NSCLC对erlotinib的敏感性^[37]^。相信随着miRNAs与肺癌耐药机制研究的进一步加深，miRNAs必然能为肺癌的诊断及治疗带来更为光明的前景!
